# Fabric Circuit Board Connecting to Flexible Sensors or Rigid Components for Wearable Applications

**DOI:** 10.3390/s19173745

**Published:** 2019-08-29

**Authors:** Qiao Li, Ziyuan Ran, Xin Ding, Xi Wang

**Affiliations:** 1 Key Laboratory of Textile Science & Technology, Ministry of Education, College of Textiles, Donghua University, Shanghai 201620, China; 2 Engineering Research Center of Digitized Textile & Apparel Technology, Ministry of Education, College of Information Science and Technology, Donghua University, Shanghai 201620, China

**Keywords:** fabric circuit board, detachable connection, flexible sensors, rigid components, wearable technology

## Abstract

Electronic textiles demand a new family of flexible circuit boards in the construction of fiber or fiber assemblies. This paper presents a stretchable woven fabric circuit board (FCB) with permanent as well as detachable electrical connections to sensors or other wearable electronics components. The woven FCB was created by integrating conductive yarns into an elastic woven fabric. Permanent connection was designed between the conductive tracks and flexible sensors; detachable connection was achieved by the helical structure of conductive yarns wrapping around the rigid component electrode encapsulated within elastomeric layer. The developed FCB, with its connections to flexible sensors or rigid components, is porous, flexible, and capable of stretching to 30% strain. The woven FCB with permanent connection to temperature sensors has a large fatigue life of more than 10,000 cycles while maintaining constant electrical resistance due to crimped configurations of the conductive track in the elastic fabric substrate and stable contact resistance. A prototype of the FCB assembly, with independent light-emitting diodes electrically linked and mechanically supported by the woven FCB, is also demonstrated for wearable applications.

## 1. Introduction

Recently, wearable technologies and applications have placed significant demands on electronic textile devices [1,2,3,4]. Instead of incorporating regular electrical components, the soft and flexible sensing elements, actuators, textile-based components, or circuit board assemblies are preferable to be embedded in ordinary apparel because they seamlessly and freely accommodate human motions and gestures for multiple purposes [5,6]. Such novel next-to-skin flexible electronics require flexible circuits or boards, known as flexible circuit boards (FCBs). FCBs augment both the design and research of fabric circuits that are not only capable of working for significant lengths of time but can endure repeated deformation in tensile, bending and shear modes [1,7,8,9] while providing stable connections for key elements even after washing.

Various types of textile circuits have been developed and reported using weaving, knitting, embroidery, as well as printing technologies [10]. Although most formed knitted textile circuits can be stretched considerably [11,12] while embroidery and printed circuits are more capable of incorporating complicated conductive patterns at a very low cost [13,14]. Of all the above technologies, woven fabric circuits have demonstrated outstanding electrical integrity due to its excellent mechanical stability [15,16]. Hence, significant attention has been paid to woven fabric circuits or FCBs [17].

One critical issue for FCBs is the achievement of a reliable electrical connection [18] between conductive tracks and other circuit, soft sensors or other electronic components [19]. To stabilize the electrical connection in the stretchable FCB systems, current solutions prefer to stiffen the soft substrate around the connection areas and create a permanent electrical connection between the conductive tracks and other electronic components. At present, most electrical connections in flexible and stretchable electronic systems [20,21,22,23,24,25] are based on different types of carriers established by soldering [26,27,28,29,30] and conductive adhesives [28,31,32], such as silver-filled epoxy [33], silicone adhesive and single-wall carbon nanotube conductive rubber paste [34], along with subsequent encapsulation of contact areas with the glob-top cover using polydimethylsiloxane (PDMS) rubber [35] or epoxy resin. However, such electrical connections introduce higher-modulus materials and weaken the flexibility, deformability, and mechanical behavior of the electronic assembly, compromising the electrical performance of stretchable electronics. Accordingly, a reliable connection between FCBs and sensors or actuators is required to ensure the service life of flexible assemblies.

Unlike FCBs, rigid electronic components and most flexible sensors or actuators have little or no resistance to washing. Hence, water should be avoided for many novel wearable electrical components to ensure the required working condition is maintained. To make a wearable textile device washable, the key electrical components should be removed during washing, which can be achieved via detachable connection. In this scenario, a detachable connection is the optimal and most potential solution to ensure both the washing ability of the whole assembly with flexible circuits and the sensing/working condition and stability of accessed electronic components. However, few studies on detachable connections for flexible circuit boards have been reported.

In this paper, a stretchable woven FCB was developed through the incorporation of selected conductive yarns into an elastic woven fabric. Stability and reliability of the FCB was evaluated. Meanwhile, practicable and reliable designs of both permanent and detachable electrical connections for the FCB were proposed, fabricated, and evaluated. Experimental results showed that the woven FCB with permanent and detachable connections to flexible sensors or rigid components was porous, soft with low elastic modulus, and capable of being stretched to 30% strain with a fatigue life of more than 10,000 cycles while maintaining electrical integrity, owing to the crimped configurations of the conductive track within elastic fabric substrate. To demonstrate a preliminary application, a prototype of an FCB assembly with detachable connections to independent light-emitting diodes (LEDs) was produced and demonstrated for the stability of connections and support in flexible electronics and wearable application.

## 2. Materials and Methods

### 2.1. Materials, Fabrication and Chacterization of Flexible Circuit Board (FCB)

Silver nanoparticles-coated polyamide yarn (SCPY for short, from Xiamen Unibest Import and Export Co., Ltd, Xiamen, China) was employed as the conductive track in the FCB. Double-covered elastic yarn consisting of spandex/polyamide filaments (provided by Zhende Medical Co., Ltd, Shaoxing, China) was used as the elastic yarn in the transverse direction. Polyamide filaments from Shanghai Kingtex Chemical Fiber Tech Co., Ltd., Shanghai, China was selected as the warp yarn in the longitudinal direction.

**Figure 1:**
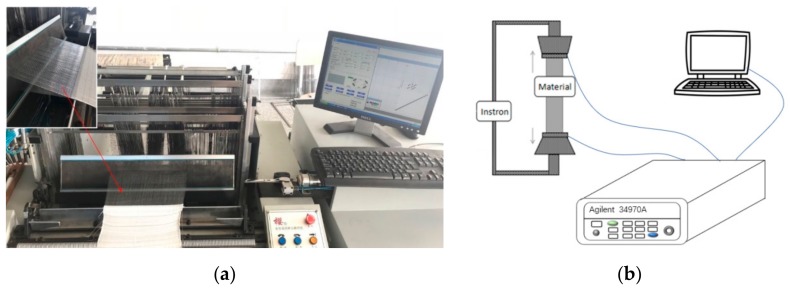
(**a**) Fabrication of stretchable woven Flexible Circuit Board (FCB); (**b**) Experimental setup for unidirectional stretching test and cyclic tensile test.

The FCB was created by weaving the SCPY into an elastic fabric comprising double-covered elastic yarn as the weft and polyamide filaments as the warp through conventional weaving technology on a semi-automatic weaving machine (Model: TNY101B, provided by Shanghai Two-Nine industry Co., Ltd., Shanghai, China), as shown in Figure 1a.

Two tests were designed and conducted, i.e., unidirectional stretching test and cyclic tensile test. As illustrated in Figure 1b, FCB samples with a size of 2.5 cm × 7 cm, gauge length of 5 cm, and an initial approximate resistance of 3.9 Ω were prepared and conditioned for strength and cyclic tensile tests. For failure test, FCB samples was stretched in transverse direction using the multifunctional strength tester (model: YG(B) 026G-500) according to standard ASTM D638. Electrical resistance of one electrically conductive track in the woven FCB was measured and recorded by Agilent 34970A. The strain rate of uniaxial tensile test was set to 400 mm/min to simulate stretching of FCB induced by normal tension induced by human movement. Strain limit of FCB was identified. For cyclic tensile test, FCB samples were stretched repeatedly within the strain range. The effect of the number of cycles of loading–unloading at given strains is to be analyzed through the analysis of the stability of the total resistance of the FCB.

### 2.2. Design and Characterization of Permanent Connections with Sensors

To test the FCB for wearable applications, the conductive track in the FCB was firstly permanently connected to the electrodes of one fabric temperature sensor previously developed by our group using a knotting and encapsulation approach. After connection, the relative resistance change of the sample with temperature was investigated under stretching conditions. A digital hot plate was used to control the temperature ranging from 25 °C to 45 °C. The resistance of the fabric sensor was recorded and acquired continuously by Agilent 34970A. Fatigue test was conducted for FCB, in which the sample was fixed to top and bottom clamps of multifunctional strength tester (YG(B) 026G-500), and two electrodes are connected to the digital multimeter (Agilent 34970A). Experimental setup was similar that mentioned above. Each cycle corresponded to a strain of 30% and then returned to original state with a tensile speed of 500 mm/min.

### 2.3. Design and Characterization of Detachable Connections

The SCPY was wrapped around a solid stainless steel needle, forming a circular helix, which was embedded into a liquid PDMS substrate, and was then cured as a whole. The solid needle was pulled out after conditioning, forming the connector holes for the electrodes of electronic components to be inserted in, which made the connection detachable. The whole woven FCB sample with resistor as external component was fixed on multifunctional strength tester (YG(B) 026G-500) and stretched in transverse direction. Electrodes of the resistor were firmly inserted into the connector holes to be introduced and connected to the circuit. Experimental setup was similar to that mentioned above. A high precision digital multimeter Agilent 34970A was implemented for resistance measurement while ramping speed of the whole sample in weft direction was 400 mm/min and 600 mm/min for cyclic loading–unloading test within 0~30% strain. The effect of insert–extraction times on the total resistance of the whole assembly was also studied.

## 3. Results and Discussion

### 3.1. Woven FCB

**Figure 2:**
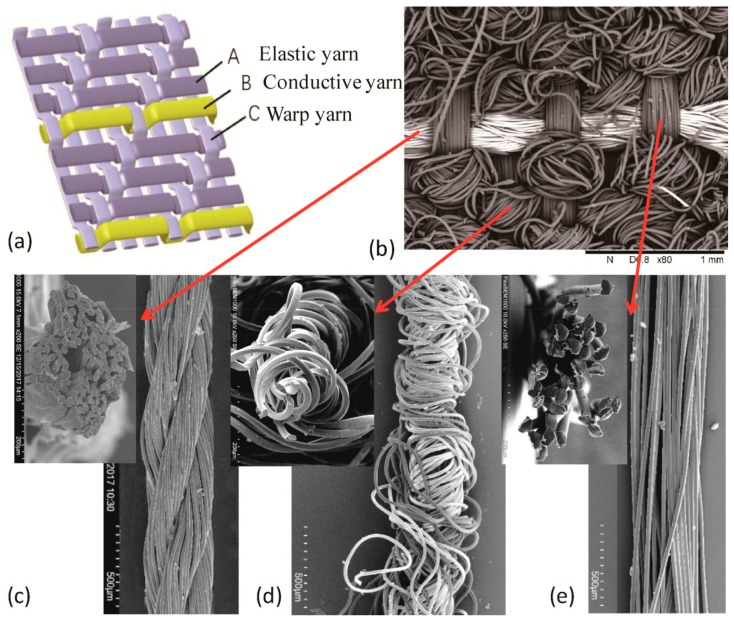
Stretchable woven FCB. (**a**) Structure of FCB; (**b**–**e**) SEM images of the woven FCB (**b**), conductive yarn (**c**), elastic yarn (**d**), and warp yarn (**e**).

The FCB was produced by incorporating conductive yarns, acting as conductive tracks, into an elastic woven fabric through conventional weaving technology on a semi-automatic weaving machine. Three different structures for FCB were realized and compared: plain, 1/2 twill, and 1/3 twill weaves. When stretched to 30% strain, the relative change of the electrical resistance of the three designs were calculated as about 291%, 188%, and 5%, respectively, due to different bucking patterns of the conductive yarn in the corresponding structures, as seen in Figure S1. Hence, the 1/3 twill weave was selected to fabricate FCB. The structure and scanning electron microscopy (SEM) images of the woven FCB with part of conductive tracks are shown in Figure 2. The FCB consists of three sets of yarns, i.e., the conductive yarn in an organized floating pattern (i.e., over one and under three warp yarns) as transverse elements (along the width direction) in the fabric, in which elastic yarns as transverse parts and polyamide yarns as longitudinal (along the length direction) elements are inter-crossed at more or less right angles to each other, as seen in Figure 2a,b. The conductive yarn used was the free-standing SCPY with a smooth surface and circular-like cross section, as seen in Figure 2c. The linear density of conductive yarn was 400 D while the electrical resistivity was measured as approximately 2 Ω·cm^−1^. The SCPY was employed as the conductive track due to its desirable mechanical flexibility (Young’s modulus of as low as 530 MPa with a Poisson’s ratio of 0.3) and robustness (tensile stress: 159.2 MPa; tensile strain: 30%); therefore, it could deform substantially to the designed crimped configurations and remain intact after incorporation into the woven structure.

The elastic yarn used in the transverse direction is the double covered elastic yarn consisting of spandex filaments with a linear density of 140 D as the core yarns and two layers of wrapping yarns of polyamide filaments with linear density of 70 D, as shown in Figure 2d. The elastic yarn could sustain stretching of up to over 200% strain and recover from 60% strain with almost no mechanical hysteresis (<5%) and stress relaxation. The warp yarn is polyamide filaments with a linear density of 210 D/32f, as seen in Figure 2e. As a result of the crimped configuration formed by relaxation of elastic yarn in the woven fabric, the FCB is capable of being stretched to a large strain, and the conductive tracks change from flexing status to straight without any over elongation. Hence, the elastic woven fabric substrate could keep the resistance of the whole circuit in a stable level under the large tensile strain limit.

**Figure 3:**
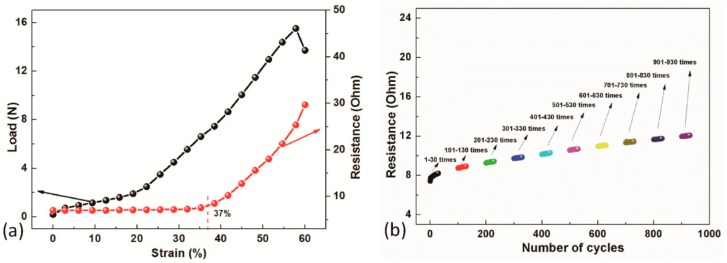
Electro-mechanical performance of the woven FCB. (**a**) Typical tensile properties of the FCB; (**b**) Typical cyclic performance of the FCB at 30% strain.

The best working strain range (especially the upper strain limit) as well as stability of electrical resistance of the FCB was evaluated. Two tests were designed and conducted, i.e., unidirectional stretching test to identify the strain limit of electrical failure and mechanical failure of the FCB and cyclic tensile test to evaluate the stability of the resistance of FCB during repeated stretching process. The SCPYs were initially unbent then stretched by the interlaced filament yarns in the tensile test. As shown in Figure 3a, electrical resistance of one conductive track in the woven FCB remained almost constant within 37% tensile strain and started to increase monotonically with strains, which is similar to the resistance-strain curve of the free-standing SCPYs, as seen in Figure S2. Additionally, there was a notable stiffness change of the FCB in tensile direction around 20% strain, possibly due to fabric structure change caused by the straightening of conductive yarns due to the stretching. The woven FCB was observed with a Young’s modulus of approximately 30 kPa in the weft direction. Figure S3 plots the electrical resistance change with different strain of the other four specimens, whose resistance remained close to stable before the critical value of 40% strain. These results suggest that the produced FCB exhibited an extraordinarily electrical stability with almost no increase in electrical resistance up to strain of 37% in unidirectional tensile test, indicating that the strain range of 0~37% is suitable for the stability of the woven FCB. Meanwhile, the stress curve indicates that the stiffness changes linearly within both the 0~20% strain and 20~60% strain range for the FCB, revealing a bi-linear mechanical behavior within 0~60% strain due to the changing of whole fabric structure at around 20% strain.

In reference to potential applications of the FCB for wearable devices and an average maximum elongation of 20~30% of the human skins, the woven FCB was then further cyclically stretched between 0~30% strain for as many as 1000 cycles. Strain rate was the same as previous test, set as 400 mm/min to simulate stretching of FCB induced by normal tension induced by human movements. The electrical resistance of the FCB changed from initial 8 Ω to about 11 Ω finally, as seen in Figure 3b, demonstrating a satisfactory stability of electrical resistance especially when incorporating high resistance components (>1000 Ω) or in low-accuracy applications.

**Figure 4:**
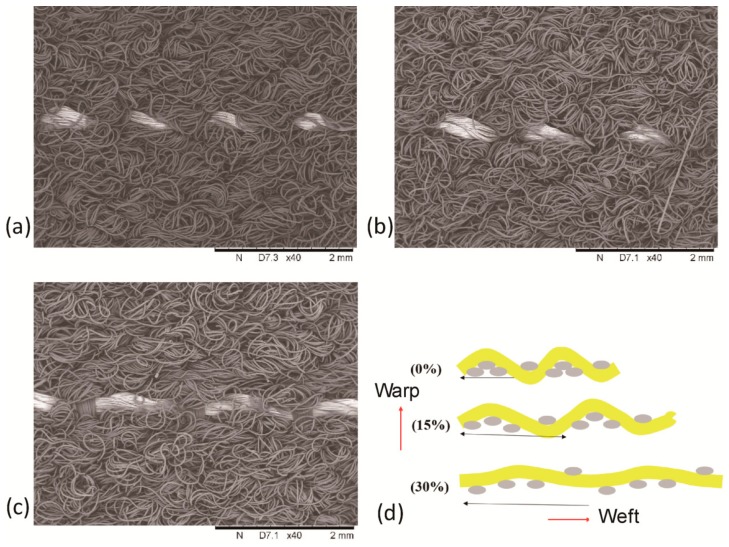
Scanning Electron Microscope (SEM) images and of the woven FCB at 0% (**a**), 15% (**b**), and 30% strain (**c**) and their corresponding schematic geometries (**d**).

To reveal the underlying mechanism that contributes to the above mechanical deformability with electrical integrity, structure morphologies of the crimped conductive tracks in the soft-woven FCB during the tensile test were examined. Morphological observation of the resultant sample was conducted on a scanning electron microscope (SEM). Figure 4a–c shows the obtained SEM images of several representative geometrical configurations of the FCB while stretched in transverse direction. During the process of stretching in transverse direction, conductive tracks were straightened and accommodated the applied tension while being straightened, with an increased period and decreased amplitude for the wavy curves, as shown in Figure 4d. At 30% strain, the yarns of conductive tracks became more dense and began to undertake tension partly, which is in accordance with the obtained 37% limit strain for stability of electrical resistance as identified above.

### 3.2. Permanent Connection to Flexible Sensors

**Figure 5:**
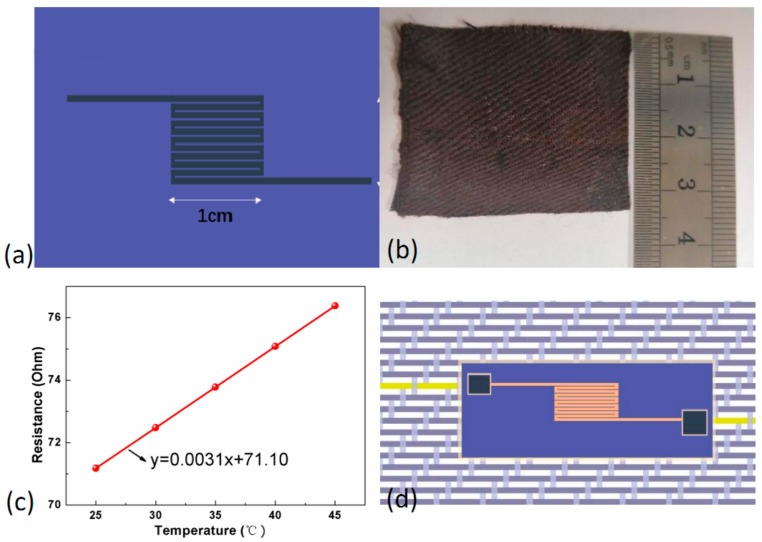
(**a**) Schematic diagram of the fabric temperature sensor; (**b**) Picture of the fabric temperature sensor; (**c**) Typical resistance– temperature curve of the fabric sensor; and (**d**) Schematic diagram of the permanent connection between the conductive track in the FCB and the sensor electrode.

To make the applications of woven FCB into wearable applications with various flexible sensors or actuators, a permanent connection between the conductive tracks and electrodes of the flexible sensors was designed, with which the electrodes of the sensors could be firmly connected to the FCB electrically. A fabric temperature sensor previously developed by our group was used as an example for demonstration. As plotted in Figure 5a,b, the temperature sensor was produced by incorporating a free-standing metal fiber platinum (Pt) with a serpentine pattern into a fabric, which was lightweight and performed like any soft and breathable garment in a non-invasive nature [17]. Figure 5c plots the typical resistance–temperature relation of the fabric temperature sensor, whose resistance rose linearly as temperature increased from 25 °C to 45 °C with a constant value of temperature coefficient of resistance (TCR), i.e., 3.1 × 10^−3^ °C^−1^, calculated by $\frac{R-R_{0}}{R_{0}(T-T_{0})}$. To connect the sensor electrodes to the conductive track in the FCB for transmission of electrical signals to outer circuits and for mechanical durability, a permanent connection was proposed based on knotting and encapsulation approach, as schematically plotted in Figure 5d. The detailed procedure is shown in Figure S4. Firstly, since the metal fiber Pt was slim, with a diameter of 20 μm, weak as well as brittle SCPY was knotted around the metal fiber gently and carefully. Secondly, a conductive silver paste (Guangzhou UV New Material Co., Ltd., China) was added to ensure the intimate contact between the sensor electrodes and the conductive track in the FCB. Finally, an encapsulated layer was created by bonding a piece of very thin fabric around the connected area to enhance mechanical robustness.

**Figure 6:**
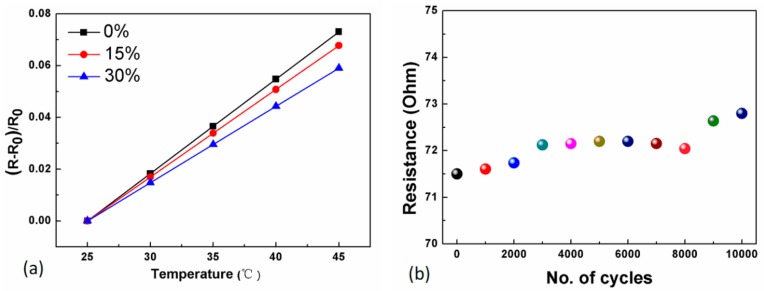
Performance of the whole assembly consisting of fabric temperature sensors connected to FCB. (**a**) Resistance-temperature relation of the fabric temperature sensor connected to the FCB; (**b**) Resistance of the fabric temperature sensor connected to FCB in cyclic stretching processes at 30% strain.

After being connected to the FCB, the electro-mechanical behavior of the fabric temperature sensor was investigated. Table 1 summarizes the electrical resistance and TCR of the fabric temperature sensors before and after being connected to the FCB at different strains. When connected to the FCB, the initial electrical resistance of the fabric temperature sensor had a slight increase in increment from 65 Ω to approximate 71 Ω due to the induced contact resistance between the conductive track and the sensor electrode; it remained almost same values while stretching the whole FCB to 15% and 30% strain, respectively. Figure 6a plots the relationship between the relative resistance change and temperature under strain of 0%, 15%, and 30%, demonstrating the reliable electrical connection between the conductive tracks and sensor electrodes, specifically, that there was no noticeable degradation in the sensitivity of the sensor with a TCR value of approximately 3.1 × 10^−3^ °C^−1^. Moreover, the fabric temperature sensor connected to the FCB was subjected to repetitive mechanical deformation of cyclic stretching at 30% strain. Figure 6b presents initial electrical resistance of the sensor when it was cyclically stretched for 10,000 cycles at 30% strain. Only a slight increment in electrical resistance was observed in the whole FCB from 71.5 Ω to 72.5 Ω after repetitive stretching test, showing that our proposed permanent connection of FCB to flexible sensors is very reliable under large deformation. Moreover, the conductive track SCPY in the FCB was kept under 60 °C and a humidity as high as 90% for 20 days. The averaged increase of resistance was observed as only about 0.5 Ω, indicating the effect of humidity on FCB is negligible, as seen in Figure S5. Hence, the effect of humidity on the whole assembly containing Pt-based temperature sensor and SCPY-based FCB is negligible as well. As a demonstration, a flexible and stretchable temperature sensing network consisting of fabric temperature sensors connected to the FCB has been produced and used for in situ monitoring of skin temperature of human bodies [17].

### 3.3. Detachable Connection to Rigid Components

**Figure 7:**
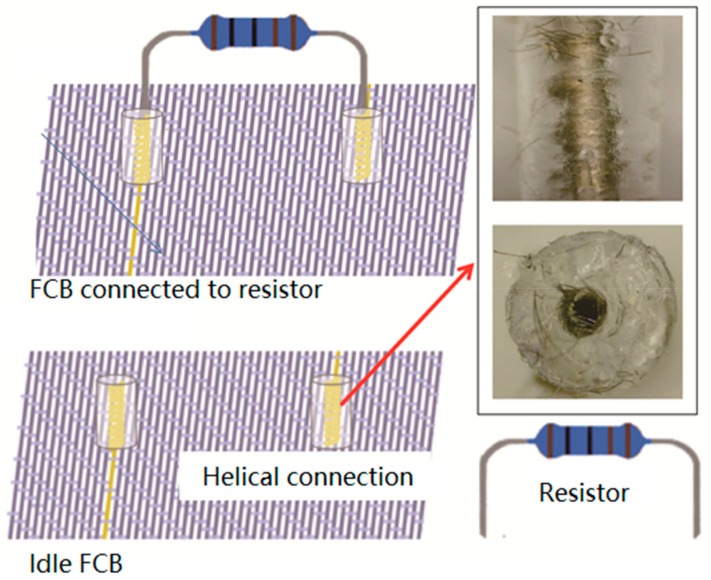
Helical connections between the conductive track and the resistor electrode.

As mentioned above, next-to-skin wearable applications based on textiles consistently demand the capability of washing or re-use. Because the reported design of FCB has demonstrated excellent washing capability over 30 times [12], a solution of detachable connection (with connector holes for electrodes) is proposed and realized to avoid damage of the conventional electronic components. To enlarge the applications of woven FCB into wearable applications with flexible or rigid electronic components, a solution of detachable connection for electronic components is proposed, with which the external electronic elements can be easily and firmly connected into the flexible circuit electrically as well as removed from the flexible circuit through insert-connectors. As illustrated in Figure 7, the electrodes of the resistors (used as a demonstration of an external electronic component) could be inserted into the connector holes of the woven FCB, by which electronic components are introduced into and motivated by the circuit. When needed, electrodes can be withdrawn from the connector holes to stop working. Most importantly, the connection of electrodes and connector holes were designed to support cyclic reuse. To realize such detachable connection setup, conductive yarn was firstly wrapped around a solid stainless steel needle with a diameter of 800 μm, the same size as that of the resistor electrodes, forming a circular helix (with outer diameter of 3 mm, inner diameter of 800 μm and height of 6 mm). The circular helix was subsequently embedded into a liquid PDMS substrate, and was then cured as a whole at 100 °C for one hour. The solid needle was pulled out after conditioning, forming the connector holes for the electrodes of externally electronic components to be inserted into, which makes the connection detachable.

**Figure 8:**
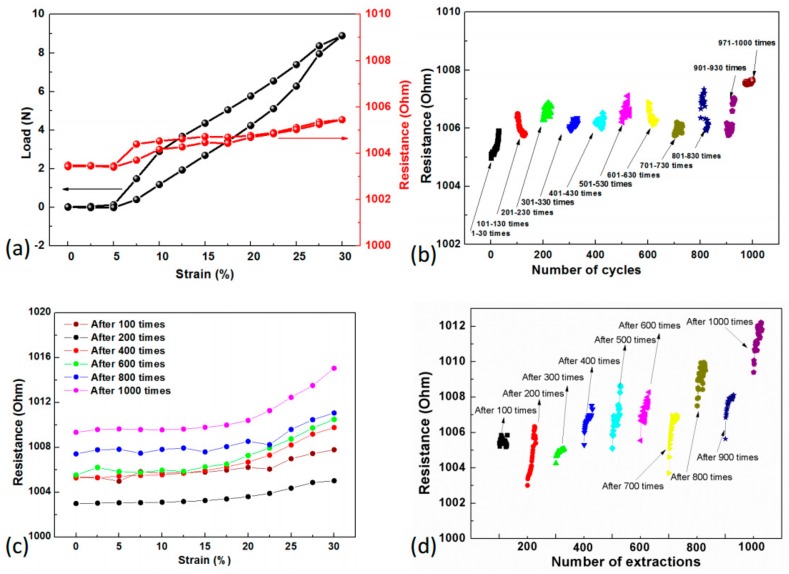
Electrical response of the helical connection in the woven FCB assembly. (**a**) Load-strain and resistance-strain relation; (**b**) Resistance variation in cyclically stretching process; (**c**) Resistance-strain relation after different times of extraction; (**d**) Resistance variation over different times of extraction.

To demonstrate the integrity of the flexible circuit, the woven FCB sample with resistor (with electrical resistance of 1000 Ω) as external component, was further fixed on multifunctional strength tester and stretched in transverse direction. Figure 8a shows that the total electrical resistance of the whole assembly increased very little, from 1003 Ω to 1006 Ω, with a relative resistance change of only 0.3% when the whole assembly was stretched to 30% strain. This result shows an excellent electrical integrity during the stretching process. It can also be observed that the 20% strain is the boundary strain level, representing the structural change of the fabric structure as the stiffness of the assembly rose after 20% strain. Figure 8b presents the effect of stretching times on the response of electrical resistance of the sample through cyclically stretching the whole sample for 1000 times within the strain range of 0~30%. It can be distinguished that the total electrical resistance generally remained around a constant value of about 1006 Ω, indicating that the woven FCB with helical connection to external components has satisfactory repeatability with an acceptable variation in electrical resistance under the safety 30% strain level, which is believed to benefit from the designed structure of the conductive track with helical patterns.

By applying the detachable connection, external components such as sensors or actuators could be pulled out from the connection holes before washing for protection. Hence, the effect of the insert–extraction times on the total resistance of the whole assembly should be studied. Figure 8c presents demonstrates that the total resistance of the FCB generally increased slowly with times of insert-extraction while being stretched within 0~30% strain. Increase of contact resistance is shown as no more than 7% after 1000 times of insert–extraction practice. Figure 8d illustrates a similar trend of increment of resistance with times of insert–extraction at fixed strain. The electrical resistance generally remained constant, implying that the resistor had an intimate and firm contact with the conductive track of the woven FCB samples during stretching. The minor overall increase in total resistance shall be addressed by the increment of contact resistance, which is believed to have been caused by diminished contact between connector holes and electrodes; the contact area between connector holes and electrodes was considerably smaller after times of insert–extraction. When connected with multimeter, there was an trend of ascending resistance of FCB; in contrast, when the FCB was not connected to multimeter, the resistance fell back to a lower value before being measured by multimeter again, revealing a ‘resistance regeneration’ phenomenon. In consideration of the setup of the test, we believe that the heat generated by contact resistance during insertion–extractions (more than by friction) provides a transient boost on the resistance of connections. Notably, when the multimeter was not used and the whole circuit was not powered on, even though the insertion–extraction continued, no heat was generated by contact resistance and the contact resistance decreased as heat slowly dissipated. Luckily, this ‘resistance regeneration’ will not affect applications of FCB since rapid insertion–extraction are not likely to occur in normal use. Such detachable design of the connections in the soft FCB assemblies can boost an increased and wider potential applications of electronic components based on various materials and technologies for multiple purposes.

### 3.4. Wearable Application

**Figure 9:**
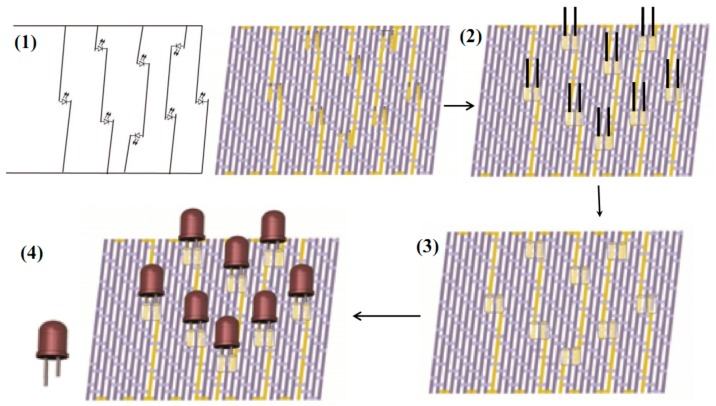
Primary processes of the FCB assembly. (**1**) Design and fabrication of the FCB according to the layout corresponding to pre-determined positions of the LEDs; (**2**) Fabrication of helical connections by wrapping SCPY around the needles with encapsulated elastomers; (**3**) Naked FCB with connection holes; (**4**) Formed FCB with inserted LEDs.

**Figure 10:**
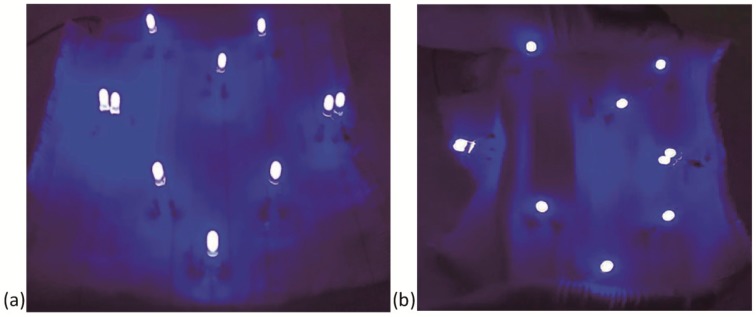
The created FCB assembly consisting of LEDs in the FCB substrate. (**a**) LEDs are illuminated at 0% strain; (**b**) LEDs remain illuminated at near 30% strain.

As a preliminary example of the above FCB and detachable connections, a simple five-way LED system designed to indicate the reliability of the FCB assembly was established based on the stretchable FCB. The LED system consisted of total eight independent LEDs physically linked to soft FCB via detachable and helical connections. The primary and schematic fabrication procedures of the LED system is shown in Figure 9. The first step involved weaving a jacquard pattern on a computerized weaving machine to define the wiring layout of the FCB corresponding to pre-designed positions of the individual LEDs. Then, the ends of the conductive tracks were wrapped around the stainless steel needles to be embedded into PDMS substrate, forming encapsulated elastomeric layer and pulled out, with connection holes left for detachable connection with external components. Finally, the LED electrodes were inserted into the connection holes of the FCB to be activated. Illumination of LEDs indicates valid connection of current conductive track. The woven FCB was connected to and driven by outer circuits or batteries by selected interposers to the conductive tracks. As illustrated in Figure 10a, all LEDs were illuminated when the whole assembly was powered by a 6V battery. Moreover, when the whole assembly was stretched by hand freely within 30% strain, all the LEDs remained illuminated, indicating the integrity and reliability of the soft FCB assemblies as well as the detachable connections, as seen in Figure 10b.

## 4. Conclusions

The rapid growth of electronic textiles demands a new family of flexible circuit boards in the construction of fiber or fiber assemblies. In this paper, the design, fabrication, and characterization of a stretchable FCB with both permanent and detachable connections between conductive tracks and flexible sensors or rigid components was proposed, realized, and evaluated. Designed for wearable electronics, the soft woven FCB was fabricated by incorporating continuous conductive yarns into an elastic fabric based on computerized weaving technologies. The manufactured woven FCBs had exhibited outstanding electrical stability under a large strain (less than 1% relative resistance change within 30% tensile strain) and satisfactory fatigue life (greater than 1000 cycles at 30% strain), which was attributed to the designed helix structure of the conductive tracks in the woven assembly. To extend applications of next-to-skin wearable technologies and electronics, simple but effective solutions of permanent and detachable connections were further proposed. In particular, the detachable connection could make sensors or external components removable for protection when washing of FCB is needed. Stable electrical resistance was observed of the FCB assembly with permanent and detachable connections when stretched at 30% strain or lower. Overall, the excellent mechanical deformability and electrical reliability of the FCB assemblies with connections to sensors or other components demonstrates the potential of wide applications in human–machine interfaces [36,37], prosthetics, and wearable skin electronics [6].

## Figures and Tables

**Table 1:** Electrical resistance and TCR of one fabric temperature sensor.

Samples	R_0_ (Ω)	R_max_ (Ω)	Temperature Range (°C)	TCR (°C^−1^)
FTS	65.65 (±0.04)	69.70 (±0.50)	25–45	0.0031
FTS_con_-0%	71.20 (±0.07)	76.41 (±0.08)	25–45	0.0037
FTS_con_-15% ^1^	71.04 (±0.03)	75.29 (±0.13)	25–45	0.0030
FTS_con_-30%	70.86 (±1.17)	75.73 (±0.37)	25–45	0.0034

^1^ FTS_con_-15% means the fabric temperature sensor connected to FCB was stretched to 15% strain.

## Supplementary Materials

The following are available online at https://www.mdpi.com/1424-8220/19/17/3745/s1, Figure S1: Relative resistance change with applied strain for different weaving structures of the FCB; Figure S2: Relative resistance change-strain relation of the SCPY; Figure S3: Resistance-strain relation of the other four specimens of the FCB; Figure S4: Fabrication procedure of the permanent connection between the conductive track and the sensor electrode; Figure S5: Resistance of the SCPY as a function of time under 60 °C temperature and 90% humidity.
