# Supplementary material for: Fabric Circuit Board Connecting to Flexible Sensors or Rigid Components for Wearable Applications
Source: Sensors (Basel). 2019 Aug 29;19(17):3745. doi: 10.3390/s19173745 (PMC6749427; doi:10.3390/s19173745)
Supplement: Supplementary file 1 [file sensors-19-03745-s001.pdf]

Fabric Circuit Board Connecting to Flexible Sensors or Rigid Components for Wearable Applications

Qiao Li[1], Ziyuan Ran[1], Xin Ding[1], and Xi Wang[2,*]

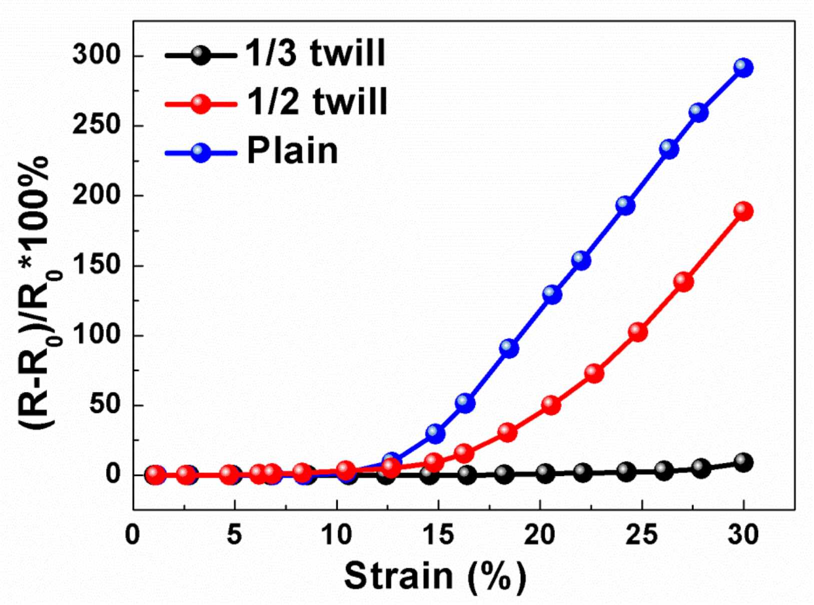

Figure S1. Relative resistance change with applied strain for different weaving structures of the FCB.

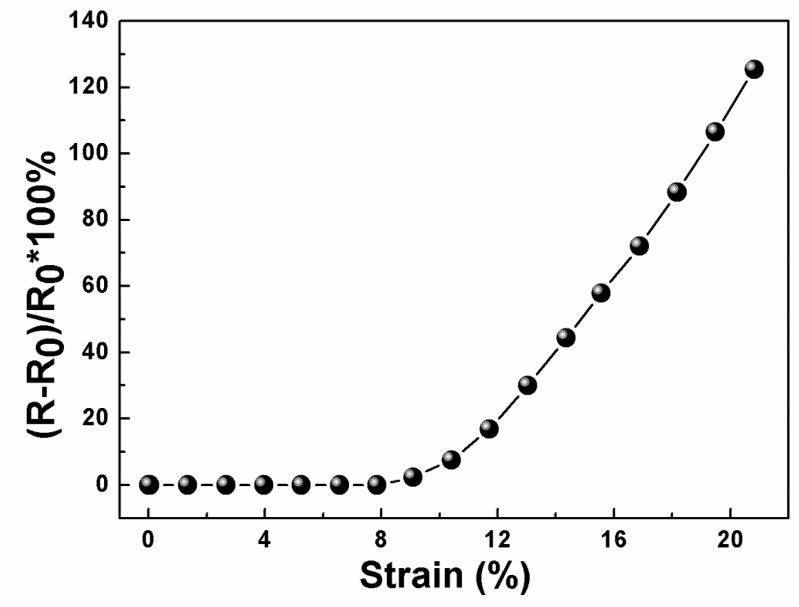

Figure S2. Relative resistance change-strain relation of the SCPY.

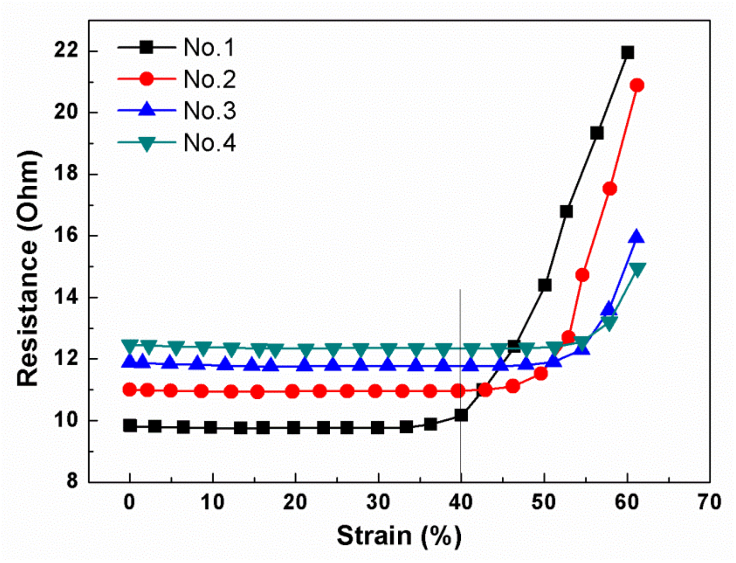

Figure S3. Resistance-strain relation of the other four specimens of the FCB.

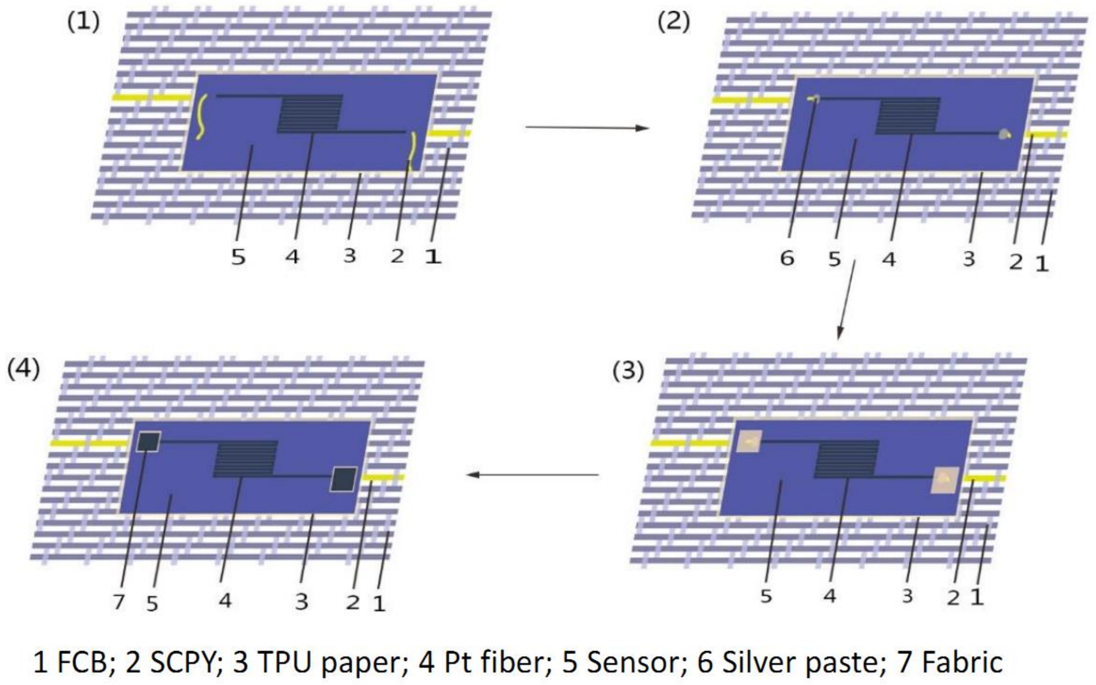

Figure S4. Fabrication procedure of the permanent connection between the conductive track and the sensor electrode.

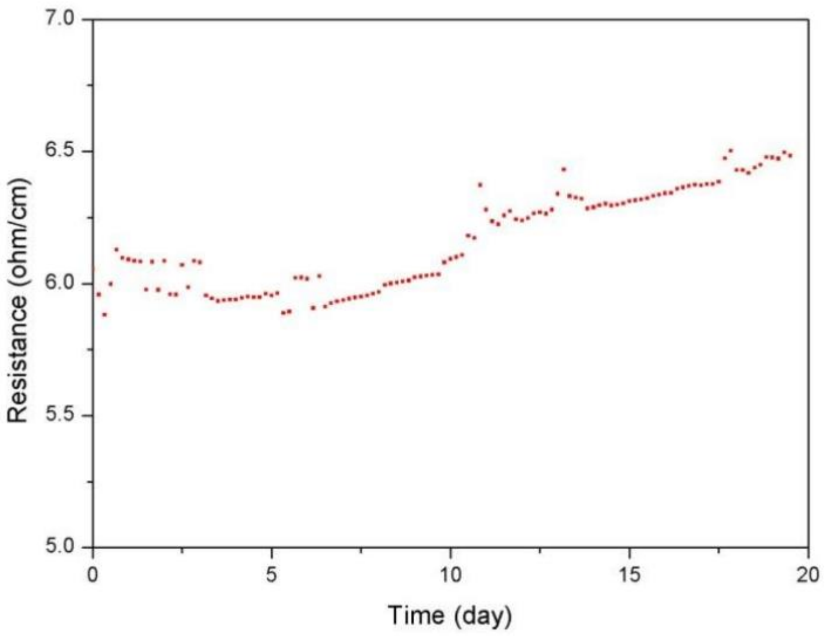

Figure S5. Resistance of the SCPY as a function of time under 60℃ temperature and 90% humidity. .
